# Ectopic Expression of Neurogenin 2 Alone is Sufficient to Induce Differentiation of Embryonic Stem Cells into Mature Neurons

**DOI:** 10.1371/journal.pone.0038651

**Published:** 2012-06-13

**Authors:** Eva C. Thoma, Erhard Wischmeyer, Nils Offen, Katja Maurus, Anna-Leena Sirén, Manfred Schartl, Toni U. Wagner

**Affiliations:** 1 Physiological Chemistry I, University of Wuerzburg, Wuerzburg, Germany; 2 Physiologisches Institut, University of Wuerzburg, Wuerzburg, Germany; 3 Department of Neurosurgery, University of Wuerzburg, Wuerzburg, Germany; Ecole Normale Supérieure de Lyon, France

## Abstract

Recent studies show that combinations of defined key developmental transcription factors (TFs) can reprogram somatic cells to pluripotency or induce cell conversion of one somatic cell type to another. However, it is not clear if single genes can define a cell̀s identity and if the cell fate defining potential of TFs is also operative in pluripotent stem cells *in vitro*. Here, we show that ectopic expression of the neural TF *Neurogenin2* (*Ngn2*) is sufficient to induce rapid and efficient differentiation of embryonic stem cells (ESCs) into mature glutamatergic neurons. *Ngn2*-induced neuronal differentiation did not require any additional external or internal factors and occurred even under pluripotency-promoting conditions. Differentiated cells displayed neuron-specific morphology, protein expression, and functional features, most importantly the generation of action potentials and contacts with hippocampal neurons. Gene expression analyses revealed that *Ngn2*-induced *in vitro* differentiation partially resembled neurogenesis *in vivo*, as it included specific activation of *Ngn2* target genes and interaction partners. These findings demonstrate that a single gene is sufficient to determine cell fate decisions of uncommitted stem cells thus giving insights into the role of key developmental genes during lineage commitment. Furthermore, we present a promising tool to improve directed differentiation strategies for applications in both stem cell research and regenerative medicine.

## Introduction

During embryogenesis, all cell types of the body arise from a small pool of stem cells by differentiation – a complex process of defined sequential steps. Transcription factors (TFs) play an important role during this process by regulating the specific gene expression program of the various stages or triggering the transition to the next step. It has been shown that the ability of such key developmental genes to influence cell fates can also be operative outside of normal physiological development. Thus, ectopic expression of three defined genes can convert pancreatic exocrine cells into ß-cells *in vivo* [1]. Similar *in vitro* experiments report the reprogramming of somatic cells to a pluripotent state [2], [3] or the *in vitro* conversion of fibroblasts into neurons by specific combinations of defined TFs [4]. Furthermore, there are reports demonstrating that ectopic expression of lineage-specific genes can influence lineage decisions of *in vitro* differentiating stem cells. For example, ectopic expression of the neural TFs *Sox1* or *Sox2* in murine embryonic stem cells (mESCs) promotes the differentiation towards the neuroectodermal lineage upon induction of differentiation [5].

All these studies indicate that key developmental genes can define a cell’s identity outside of their physiological context. However, it is not clear if this cell fate defining potential depends on additional external signals or is only operative in certain cell types. Cell fate conversion by ectopic expression of certain genes *in vivo* is always performed on at least partially committed cells. Additionally, such processes may be influenced by unknown factors of the *in vivo* environment. *In vitro* cell conversion or reprogramming protocols are also performed with differentiated cells and generally include the addition of specific culture media components to enhance formation or survival of the desired cell type. Similarly, in reports describing directed differentiation of pluripotent stem cells by single defined TFs, differentiation itself was still induced and promoted by external signals like embryoid body formation or the addition of specific differentiation media [5]–[7].

Thus, until now, cell fate determination by key developmental TFs always includes the involvement of additional known or unknown factors. This makes it difficult to evaluate the strength of the cell fate defining potential of such genes.

Here, we wanted to investigate if a single key developmental gene is able to determine the cell fate of pluripotent stem cells without the need for any other external differentiation-inducing or lineage-promoting signals. We focused on the formation of neuronal cells types, as this differentiation pathway is of great interest for many applications like potential clinical therapy of neurodegenerative diseases or drug screening. Neuronal differentiation of stem cells also offers a valuable tool to study neurogenesis *in vitro* as the development of the mammalian nervous system is hardly accessible for studies of the *in vivo* situation. Here, we show that ectopic expression of the neuronal basic helix-loop-helix transcription factor *Neurogenin2* (*Ngn2*) is sufficient to induce and promote neuronal differentiation of mESCs towards the appearance of mature, functional neurons. *Ngn2*-mediated differentiation is fast and efficient and occurs even in the presence of external pluripotency-promoting signals like serum, leukemia inhibitory factor (LIF), or inhibitors of defined signaling pathways. Furthermore, our results indicate that *Ngn2* induces a specific neuronal differentiation process that is – in certain aspects - reminiscent of the corresponding *in vivo* situation.

## Materials and Methods

### Plasmids

The *Ngn2* expression construct was a kind gift from F. Guillemot and contains the coding sequence of *Ngn2* with a N-terminal myc tag under control of the CMV promoter. As a transfection control, cells were co-transfected with pEGFP(C1)-Zeo, a vector coding for a fusion protein of the fluorescent protein EGFP and the zeocin resistance under control of the CMV promoter. Ratios were 1.5 µg expression vector +0.5 µg pEGFP(C1)-Zeo. Control cells were transfected with EBFP-N1 (kind gift of R. Campbell, P. Daugherty, and M. Davidson) instead of the *Ngn2* expression construct.

For generation of the *Ngn2* induction constructs the pminiTol2/MCS vector (kind gift from S. Ekker), that contains the tol2 recognition sites, was modified by inserting a CMV promoter and a polyA tail resulting in the vector pMTCpA. For generation of the P2Angn2 construct the EGFP-Zeo coding sequence was amplified by PCR with primers containing flanking lox sites. PCR product was inserted in pMTCpA resulting in pMTC-EGFP-Zeo. Subsequently, the CMV promoter was replaced by ef1a1 promoter resulting in pMTE-EGFP-Zeo. Then, the coding sequence of *Ngn2* was amplified by PCR and inserted in pMTE-EGFP-Zeo resulting in pMTE-EGFP-Zeo-ngn2. Puromycin resistance gene and 2A sequence were amplified by PCR and cloned into pMTE-EGFP-Zeo-ngn2 resulting in the final P2Angn2 construct.

For generation of the CreP2Angn2 construct the coding sequence of CreERT2 linked to a 2A sequence was amplified by PCR and cloned into pMTE-EGFP-Zeo-P2Angn2.

### Cell Culture

Mouse ES cells were a subclone of an established ESC line originally named E14 [8]. ESCs were grown at 37°C, 5% CO_2_ on gelatin coated wells in DMEM with stable glutamine (Invitrogen, 4 mM), 10% FBS, sodium pyruvate (1 mM), non-essential amino acids (0.1 mM), penicillin/streptomycin, ß-mercaptoethanol (0.1 mM), and LIF (1000 U/ml). For all experiments in serum-containing medium, the same batch of fully defined FBS (PAA) was used. For 2i medium culture conditions, cells were transferred to Knock-out DMEM (Invitrogen) supplemented with knock-out serum replacement (Invitrogen), stable glutamine (Invitrogen, 4 mM), penicillin/streptomycin, non-essential amino acids (0.1 mM), sodium pyruvate (0.1 mM), ß-mercaptoethanol (0.1 mM), LIF (1000 U/ml), PD0325901 (StemGent, 1 µM), and CHIR99021 (StemGent, 3 µM).

During all differentiation experiments, medium was changed every day. If differentiation was performed in the absence of LIF, cells were transferred to stem cell medium without LIF beginning from the day of transfection/recombination.

Transfections were performed using the Fugene HD transfection reagent (Roche) following the manufacturer’s instructions. Transfection was performed for 24 hours. Total amounts of transfected DNA were 2 µg per 6-well, 1 µg per 12 well and 0.6 µg per 24 well. Transfection efficiency estimated by expression of cotransfected fluorescent proteins was about 40–50 percent. Transiently transfected cells were selected with zeocin (100 µg/ml) starting at 2 days post transfection (dpt).

For electrophysiology, E14-P2Angn2 cell line was used that allows induction of *Ngn2* expression and the puromycin resistance gene by addition of Cre recombinase as transducible protein.

For generation of E14-P2Angn2 and E14-CreP2Angn2 cell line, the tol2 transposase system [9] was used. Cells were transfected with a construct containing the coding sequence of the tol2 transposase under control of the CMV promoter and the P2Angn2 construct or the CreP2Angn2 construct, respectively, at ratio of 2∶1. Subsequently, cells were grown at very low density and selected with zeocin (100 µg/ml) for 10 days to allow the formation of single colonies. Single colonies were picked manually under a microscope, expanded, and checked for correct function of the induction constructs. For each cell line, one clone was chosen for further analysis.

For protein transduction, E14-P2Angn2 cells were seeded as single cell suspension on gelatin coated wells. After cell adhesion, cells were treated with serum-free stem cell medium containing 2.5 µM TATCre recombinase [10] (kind gift from F. Edenhofer) for 4 hours. Then, cells were kept in LIF-free, serum-containing medium.

For induction of *Ngn2* expression in E14-CreP2Angn2 cell line, CreERT recombinase [11] was activated by treating the cells with 4-hydroxytamoxifen (4OHT). 4OHT stock solution (1 mM, dissolved in 100% ethanol) was diluted 1∶1000 in cell culture medium to a final concentration of 1 µM. Mock cells were treated with the same volume of 100% ethanol. After 18 hours, medium was changed for both 4OHT and mock treated cells. Medium consisted of complete ESC growth medium with and without LIF, respectively. For combination of medium-based and *Ngn2*-induced differentiation, cells were transferred to N2B27 medium (1∶1 DMEM/F12 and Neurobasal supplemented with N2, B27 and penicillin/streptomycin) after 4OHT treatment.

For both E14-P2Angn2 and E14-CreP2Angn2 cell line, puromycin selection was started 2 days post recombination (dpr) at a concentration of 1 µg/ml and raised to 2 µg/ml 5dpr. Puromycin selection was continued until the end of the experiments.

For, coculture experiments, E14-CreP2Angn2 cells were seeded on poly-D-lysine coated coverslips and *Ngn2* expression was induced by treatment with 4OHT. Differentiating cells were cultured in serum-containing stem cell medium without LIF and selected with puromycin. Medium was changed every day. 8dpr, cells were labeled with CellTracker™ Green CMFDA (Invitrogen) according to the manufacturer’s instructions and cultured together with hippocampal neurons from C57Bl/6 mouse fetuses at E18 (breeding pairs from Harlan-Winkelmann, Borchen, Germany) in MEM with 2% B27, 0.22% sodium bicarbonate, 1 mM sodium pyruvate, 2 mM L-glutamine, 1% Penicillin/Streptomycin, and 0.6% glucose. Cultures were incubated at 37°C under 5% CO2/95% air and 90% humidity for 12 days. Medium was changed every second day.

### RT-PCR

Total RNAs were isolated from cell cultures using the Total RNA Isolation Reagent (AB Gene). Samples were digested with DNAseI (Fermentas) to exclude gDNA contamination followed by cDNA synthesis (Fermentas).

Polymerase chain reactions from 25 ng cDNA were run with the following primers: Ef1a1 5′- GGTGACAACATGCTGGAGCCAAGTG-3′, 5′-CCCACAGGGACAGTGCCAATGC-3′; dcx 5′-CCATTGACGGATCCAGGAAG-3′, 5′-TCTGGCTTGAGCACTGTTGC-3′; math3 5′-GCCCAGAGACTGTGGTACTGA-3′, 5′-AGAGCCCGGTCTTCTCTCTT-3′; neuN 5′-AGGACTACTCCGGCCAGACC-3′; 5′-TAGTCGTTTGGGCTGCTGCT-3′, endogenous ngn2 5′-GACATTCCCGGACACACACC-3′, 5′-CTCCTCGTCCTCCTCCTCGT-3′; olig2 5′-ACAGACCGAGCCAACACCAG-3′, 5′-CGGGCAGAAAAAGATCATCG-3′; pax6 5′-GAAGCGGAAGCTGCAAAGAA-3′, 5′-GGAGTGTTGCTGGCCTGTCT-3′; sox1 5′-GCTGCAGTACAGCCCCATCT-3′, 5′-GGCTCCGACTTGACCAGAGA-3′; vGLUT1 5′-CGCTTGTTTCTGCCTGTGTG-3′, 5′-TGGTTAGGCGAGCCTTGAAA-3′; vGLUT2 5′-CAATTTAAATCTGGTAAGGCTGG-3′, 5′- CCTTCTTCTCAGGCACCTC-3′; transient ngn2 5′-TCGCCCGCTAGCCCCGGGTC-3′, 5′-CAAGCGGCTTCGGCCAGTAACGTTA-3′; inducible ngn2 5′-GTGCATGACCCGCAAGCCCG-3′, 5′-CTCCTCGTCCTCCTCCTCGT-3′; th 5′-GGCTTCTCTGACCAGGCGTA-3′, 5′-TCCTCCAGCTGTGGAATGCT-3′; gad1 5′-GCGCACAGAGACCGACTTCT-3′, 5′-CTTCCATGCCTTCCAGCAAC-3′; oct4 5′- CACGAGTGGAAAGCAACTCA-3′, 5′- AGATGGTGGTCTGGCTGAAC-3′, nanog 5′- AAGTACCTCAGCCTCCAGCA-3′, 5′- GTGCTGAGCCCTTCTGAATC-3′; afp 5′- CTCAGCGAGGAGAAATGGTC-3′, 5′- GGTGATGCATAGCCTCCTGT-3′, insulin 5′- ATTGTTTCAACATGGCCCTGT -3′, 5′- CTTGTGGGTCCTCCACTTCAC -3′; myoD1 5′- CTACGACACCGCCTACTACAGTGA-3′, 5′- CCTCTGCTGCTGCAGTCGATCT-3′, PU1 5′- CTCCATCAGACACCTCCAGGGG-3′, 5′- CAGCTACAGCAGCTCTATCGCC-3′.

For positive controls, cDNAs from the following tissues or cell lines were used: brain for neuroectodermal markers, embryonic stem cells for pluripotency markers, liver for endodermal markers, muscle and liver for mesodermal markers.

Quantitative RT-PCR was performed from 25 ng cDNA for 40 cycles with SYBR Green reagents and amplifications were detected with a Biorad-iCycler. Ef1a1 primers were used for template normalization. Each reaction was performed in triplicate. Data from three independent experiments were analysed and the average value +/− SD is shown. To evaluate significance, Student’s t-test was performed.

### Immunofluorescence

Immunofluorescence staining was performed as described [12] using anti-Tuj1 antibody (Novus Biologicals, 1∶1000), anti MAP2ab antibody (Acris Antibodies GmbH, 1∶250), anti-Nanog-antibody (antikoerper-online, 1∶1000), anti-Stat3 antibody (Santa Cruz, 1∶1000), anti-vGlut1 antibody (SynapticSystems, 1∶1000), anti-tau antibody (SynapticSystems, 1∶1000), anti-NR1 antibody (Sigma, 1∶1000), anti-Synapsin 1 (SynapticSystems, 1∶1000), anti-MAP2 (Chemicon, 1∶500), anti-Tuj1 (R&D systems, 1∶600), anti-th (Sigma, 1∶500), anti-myc (cell signalling, 1∶2000), Hoechst 33258 (Molecular probes), and DAPI (Sigma).

For staining of coculture experiments, 10% normal horse serum (NHS) instead of 5% BSA was used for blocking.

### Quantification Experiments

For all experiments, an equal number of cells were seeded for *Ngn2* and mock treatment.

To determine efficiency of neural differentiation after transient transfection, Tuj1 positive cells were counted in an area of 15.04 mm^2^. Data from three independent experiments were evaluated using Student’s t-test and Figures display mean +/− SD.

To determine efficiency of neural differentiation in E14-CreP2Angn2 cells, total cells and Tuj1 positive cells were counted in an area of 3 mm^2^. Data from three independent experiments were evaluated using Student’s t-test and Figures display mean +/− SD.

For quantification of Nanog expression 3dpr, total cells and Nanog positive cells were counted in six to eight representative stem cell colonies for mock and 4OHT treated cells. Data from three independent experiments were evaluated using Student’s t-test and Figures display mean +/− SD.

### Microscopy

Light microscopy was performed using a Leica DMI6000B inverted microscope. Confocal microscopy was performed using either a Nikon C1 confocal microscope or a SP5 Confocal Microscope (Leica). All images were analysed using ImageJ software.

### Electrophysiology

Whole-cell recordings [13] were performed at room temperature in a bath solution consisting of 125 mM NaCl, 2.5 mM KCl, 2 mM CaCl_2_, 1 mM MgCl_2_, 10 mM HEPES, pH 7.4. Patch pipettes were pulled from borosilicate glass capillaries (Kimble Products, England), and heat-polished to give input resistances of 3–7 MΩ (pipette resistance, whole-cell). The pipette recording solution contained 140 mM KCl, 2 mM MgCl_2_, 0.01 mM CaCl_2_, 1 mM ethylene-bis(oxyethylenenitrilo) tetraacetate (EGTA), 1 mM Na_2_ATP, 0.1 mM cyclic AMP, 0.1 mM ATP and 5 mM HEPES (pH 7.3, adjusted with KOH). Currents were recorded with an EPC9 (Heka) patch clamp amplifier and low pass-filtered at 1–2 kHz. Stimulation and data acquisition were controlled by the PULSE/PULSEFIT software package (Heka) on a Macintosh computer, and data analysis was performed with IGOR software (WaveMetrics, Lake Oswego, USA).

## Results

### *Ngn2* is Sufficient to Induce Neuronal Differentiation

Murine ESCs were transiently co-transfected with a myc-tagged *Ngn2* expression construct and GFPzeo, a fusion protein of GFP and the zeocin resistance protein allowing both visualization and selection of transfected cells. LIF, which prevents differentiation of mESCs under standard growth conditions, was withdrawn from the medium. Apart from LIF removal and zeocin selection, no other changes to either the medium or growth conditions were performed. Five days post transfection (dpt), cells with neuronal morphology could be detected in *Ngn2*-transfected cultures. These cells expressed pan-neuronal proteins Tuj1 and Map2ab (Fig. S1A-D) suggesting the formation of neurons upon *Ngn2* transfection. Immunofluorescence staining using anti-myc-Tag antibody confirmed that Tuj1+ cells expressed ectopic *Ngn2* (Fig. S2A-C).

To investigate the process of differentiation at the molecular level, we analyzed the gene expression pattern of the *Ngn2*-transfected cells 5 and 7dpt and compared it to that of untreated and mock-transfected ESCs (Fig. S1K). We focused on the typical pan-neural marker genes *Math3*, *Olig2*, and *Sox1,* which are all known to be activated early during neuronal development [14]–[16]. Furthermore, different studies show that each of these genes plays an important role during the *in vivo* development of functional neurons as loss of function always results in severe neuronal defects [17]–[19]. Moreover, expression of the late neuronal marker genes *Dcx* and *NeuN* was analyzed. During embryonic neurogenesis, *Dcx* is expressed in migrating early postmitotic neurons [20], [21] and *NeuN* in terminally differentiated postmitotic neurons [22]. All investigated genes were clearly upregulated in the *Ngn2*-transfected cells and were either not transcribed or only at background levels in mock-transfected cells. Furthermore, *Ngn2*-transfected cells showed a weak induction of endogenous *Ngn2* at both 5 and 7dpt. This expression profile confirms at a molecular level the induction and progression of neural differentiation upon *Ngn2* transfection.

To determine the efficiency of *Ngn2*-induced differentiation and to exclude the possibility of neurons arising due to random differentiation, we determined the number of Tuj1 positive cells in a wide field scan of *Ngn2*-transfected versus mock-transfected cells. After *Ngn2* transfection, a significantly higher number (p = 0.0046 (5dpt), p = 0.0012 (7dpt)) of Tuj1 positive cells was detected compared to mock-transfected cells (Fig. S1L).

Next, we wanted to investigate if ectopic expression of *Ngn2* induces the loss of stem cell identity which is a typical step during the early phase of differentiation processes. Immunofluorescence staining for the stem cell marker Nanog [23], [24] 3dpt revealed a loss of Nanog expression specifically in *Ngn2*-transfected cells (Fig. S1E-J).

### *Ngn2*-mediated Differentiation is Not Counteracted by Pluripotency-promoting Signals

Hitherto, the data indicated that transient expression of *Ngn2* is able to induce neuronal differentiation. Next, we wanted to determine the inductive strength of the signal mediated by *Ngn2*. Thus, mESCs were transfected with *Ngn2* and, for the full duration of the following experiments, were kept in adherent culture in complete ESC medium containing serum and LIF. To confirm the presence of active LIF signaling, non-transfected mESCs were treated with conditioned medium from *Ngn2*-transfected cells and stained for STAT3, a known effector of LIF signaling [25] (Fig. S3). The staining showed a clear nuclear localization of STAT3 confirming the presence of active LIF in the medium of *Ngn2*-transfected cells.

**Figure 1:**
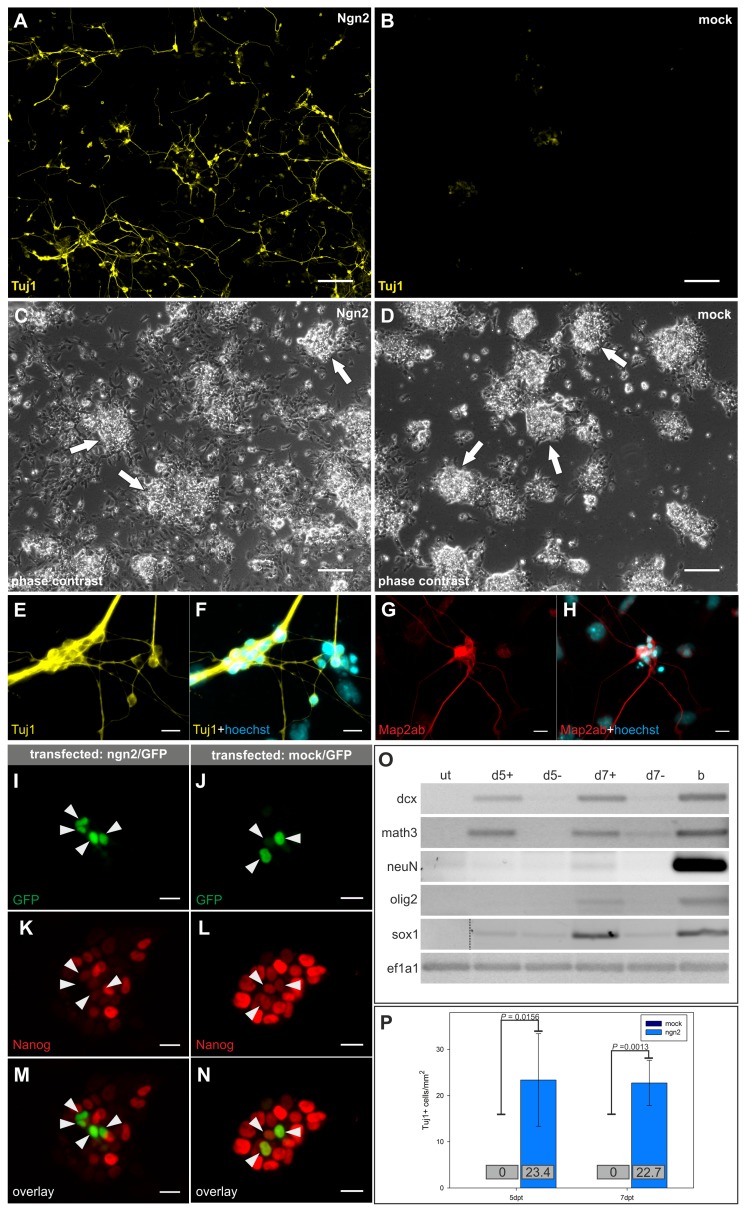
Induction of neuronal differentiation by transient transfection with *Ngn2* in complete stem cell growth medium with LIF. (A-D) Wide field scans of Tuj1 staining 5 days post transfection (dpt). Dark field view (A, B) showing Tuj1 positive cells in *Ngn2*-transfected cells (A), but not in mock-transfected cells (B). Corresponding phase contrast images (C,D) reveal the presence of stem cell like colonies (arrows). Scale bars: 200 µm. (E-H) Close-up views of developing neurons 5dpt expressing Tuj1 (E,F) and Map2ab (G,H). Scale bars: 20 µm. (I-N) Loss of Nanog expression (arrowheads) 3dpt in *Ngn2*-transfected (I,K,M), but not in mock-transfected cells (J,L,N). Transfected cells are visualized by expression of cotransfected GFP (I,J). Scale bars: 20 µm. (O) Gene expression pattern of untreated (ut), *Ngn2*-transfected (d5+,d7+), and mock-transfected (d5-,d7-) mESCs 5 and 7dpt. b: Brain cDNA. Dashed line indicates grouping of different parts from the same gel. A representative result from three independent experiments is shown. (P) Tuj1 positive cells in *Ngn2*-transfected and mock-transfected cells 5 and 7dpt. Absolute numbers are shown as non-differentiating cells continue proliferating. Therefore, the relative number would not really reflect the increase of neurons upon *Ngn2* compared to mock transfection. Mean numbers +/− SD of three independent experiments are shown.

Surprisingly, even under these pluripotency-supporting conditions, *Ngn2*-transfected cells adopted a neuronal morphology and expressed Tuj1 and Map2ab within 5dpt (Fig. 1A-H). Immunofluorescence staining for myc-Tag confirmed that Tuj1 positive cells expressed ectopic *Ngn2* (Fig. S2D-F). Mock-transfected cells did not show any specific Tuj1 staining and continued to grow in stem cell like colonies (Fig. 1B, 1D). Quantification of Tuj1 positive cells confirmed that a significantly higher number of neurons (p = 0.0156 (5dpt), p = 0.0013 (7dpt)) was detected in *Ngn2*-transfected cells compared to mock-transfected cells (Fig. 1P). Moreover, even in the presence of LIF, loss of Nanog expression was detected specifically in *Ngn2*-transfected cells (Fig. 1I-N).

Analysis of the gene expression pattern of *Ngn2*-transfected cells in LIF-containing medium led to similar results compared to cells differentiated in the absence of LIF: Early and late neural marker genes were upregulated (Fig. 1O) except for endogenous *Ngn2* which could neither be detected in *Ngn2*-transfected nor in mock-transfected cells (data not shown).

### Establishment of an Inducible *Ngn2* Expression System for Efficient and Homogenous Neural Differentiation

**Figure 2:**
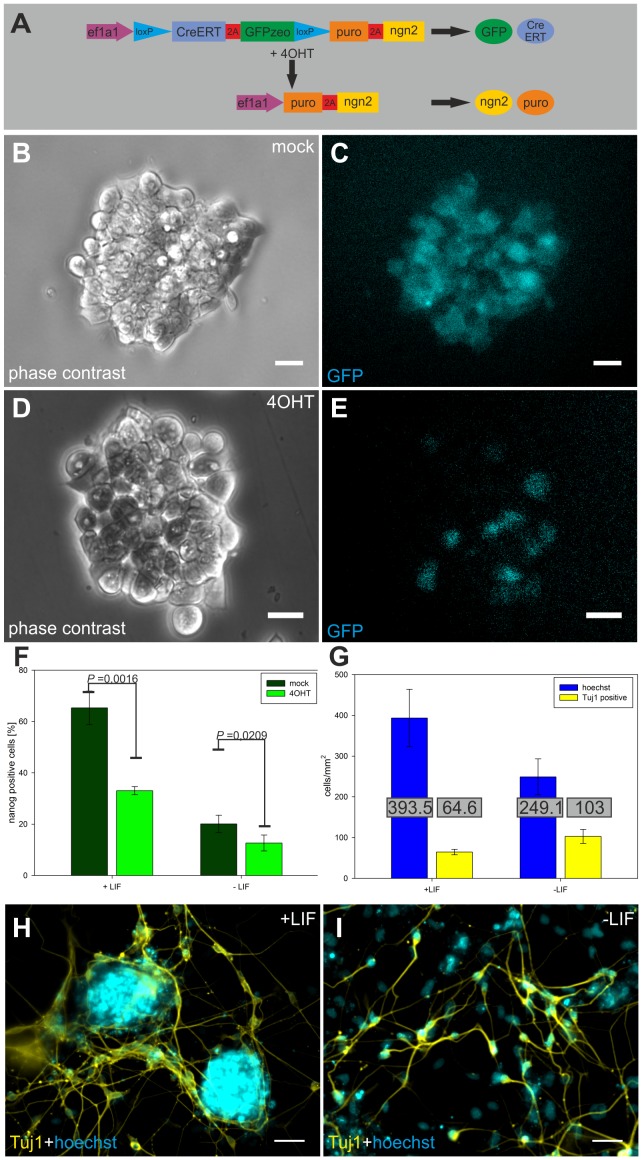
Directed neuronal differentiation using E14-CreP2Angn2 cell line. (A) Inducible construct of E14-CreP2Angn2 cell line and method of induction. (B-E) Loss of GFP signal in 4OHT treated cells (D,E) 1 day post recombination (dpr) (B,C) Mock treated cells. Scale bars: 20 µm. (F) Nanog positive cells in mock and 4OHT treated cells 3dpr in the presence and absence of LIF. Columns represent mean +/−SD of three independent experiments. (G) Numbers of total and Tuj1 positive cells 7dpr in the presence and absence of LIF. Mean numbers +/− SD of three independent experiments are shown. (H, I) Tuj1 staining 7dpr of cells grown in the presence (H) or absence (I) of LIF. Nuclei are visualized by Hoechst staining. Scale bars: 50 µm.

A differentiation system based on transient transfection has several disadvantages: Differentiation directly correlates with transfection efficiency and probably with copy number of transfected plasmids per individual cell. Furthermore, complete elimination of non-transfected cells is difficult. To overcome these problems, we generated a clonal transgenic mESC line (E14-CreP2Angn2) allowing induction of *Ngn2* and subsequent selection of *Ngn2*-expressing cells. Upon activation of CreERT by tamoxifen treatment, GFPzeo expression is replaced by expression of *Ngn2* and the puromycin resistance gene (Fig. 2A-E). Thus, by allowing the generation of a homogenous culture of stem cells expressing *Ngn2* the E14-CreP2Angn2 cell line enables a detailed analysis of the *Ngn2*-induced differentiation process.

First, loss of stem cell identity upon *Ngn2* induction was analysed (Fig. 2F, Fig. S4). Three days post recombination (3dpr), Nanog positive cells represented about 65 percent in control cells in the presence of LIF. This result is in agreement with previous studies showing a fluctuation of Nanog expression in mESCs [26]. Induction of *Ngn2* expression resulted in a significant decrease of Nanog positive cells (33 percent). Likewise, in the absence of LIF, the number of Nanog positive cells was significantly reduced in *Ngn2* expressing cultures compared to control cultures (Fig. 2F, Fig. S4).

**Figure 3:**
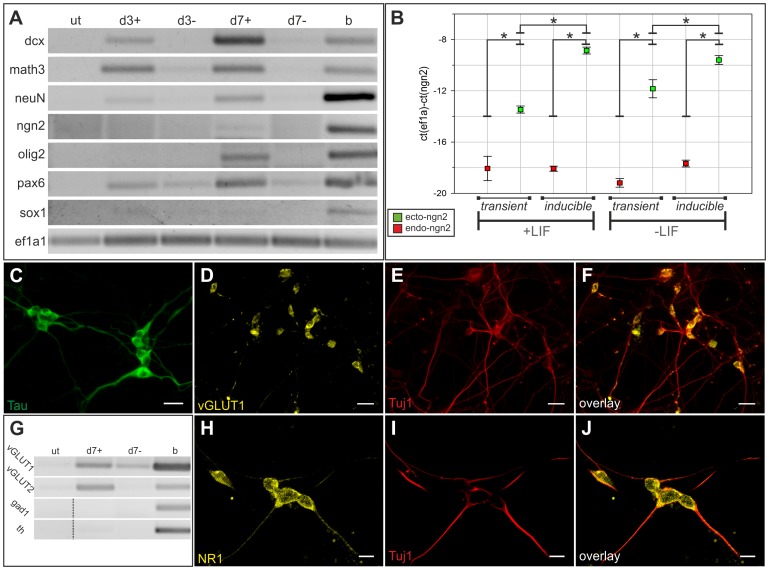
Characterization of differentiation process of E14-CreP2Angn2 cell line in the absence of LIF. (A) Gene expression pattern of untreated (ut), 4OHT treated (d3+,d7+), and mock treated (d3-,d7-) mESCs 3 and 7dpr. b: Brain cDNA. A representative result from three independent experiments is shown. (B) Expression levels of ectopic and endogenous *Ngn2* after transient transfection and in E14-CreP2Angn2 cell line at day 7 of differentiation in the presence or absence of LIF. Boxes show mean +/−SD of three independent experiments. *: p<0.01. (C) Expression of neuronal protein Tau. Scale bar: 20 µm. (G) Expression analyses of neuronal subtype markers *vGLUT1*, *vGLUT2*, *Gad1*, and *Th*. Dashed lines indicate grouping of different parts from the same gel. A representative result from three independent experiments is shown. (D-F, H-J) Expression of vGLUT1 protein, and NMDA receptor 1 (NR1) indicating formation of glutamatergic neurons. Scale bars: 20 µm (D-F), 10 µm (H-J).

Next, we analysed whether induction of *Ngn2* resulted in neuronal differentiation. Seven dpr, Tuj1 positive cells with neuronal morphology were detected in both LIF-free and LIF containing conditions (Fig. 2H, 2I). Tuj1 positive cells represented about 40 percent of total cells in the absence of LIF and about 16 percent in the presence of LIF (Fig. 2G, Fig. S5A). The lower percentage observed in the presence of LIF resulted from a higher number of total cells. Possibly, LIF promoted the higher proliferation of cells that did not respond to *Ngn2* expression by neuronal differentiation. This interpretation is also supported by the fact that - in the presence of LIF - differentiating cultures still contained stem cell-like colonies (Fig. 2H). Immunofluorescence staining confirmed that these colonies predominately consisted of Nanog+/Tuj1- cells (Fig. S5B-D). To further determine the fate of cells that did not form neurons upon *Ngn2* induction, the expression of pluripoteny markers (*Nanog, Oct4*), mesodermal (*MyoD1, PU.1*) and endodermal markers (*Afp, Insulin*) was analysed (Fig. S5E). These data revealed that 7dpr both in LIF-free and LIF-containing conditions, mRNA of pluripotency markers was still detectable with only a slight downregulation of *Nanog* in LIF-free conditions. Meso- and endodermal markers, however, were not specifically upregulated in 4OHT-treated cultures as it was the case for neuronal markers (Fig. 3A). This indicates that cells expressing ectopic *Ngn2* either remained in a pluripotent state or underwent neuronal differentiation.

Next, we wanted to analyse in detail the neuronal differentiation process occurring in E14-CreP2Angn2 cells. Gene expression pattern of the resulting neurons differentiated without LIF was highly similar to that observed in transient transfection experiments. Early and late neuronal marker genes were activated, albeit *Sox1* was not upregulated. Additionally, we detected activation of the early neural marker *Pax6* [27] (Fig. 3A). In the presence of LIF, E14-CreP2Angn2 derived neurons showed a similar gene expression pattern with upregulation of *Dcx, Math3, NeuN, Olig2,* and *Pax6* (Fig. S6).

Quantitative real-time PCR was performed to determine the expression levels of ectopic and endogenous *Ngn2* in both transient transfection assays and in the inducible cell line. In both setups, endogenous *Ngn2* was virtually not expressed. Expression levels of ectopic *Ngn2* at day 7 were significantly higher in the inducible cell line compared to transient transfection assays. The presence of LIF did not influence the levels of ectopic or endogenous *Ngn2* (Fig. 3B).

More detailed characterization of differentiated cells showed that *Ngn2*-derived neurons stained positive for Tau (Fig. 3C). Furthermore, expression of vesicular glutamate receptors *vGLUT1* and *2* mRNAs was detected while markers for other neuronal subtypes like *Tyrosine hydroxylase* (*Th*) and *Gad1* were not expressed (Fig. 3G). On protein level, cells were positive for vGLUT1 and NMDA receptor 1 (NR1) (Fig. 3D-F, 3H-J) indicating the formation of glutamatergic neurons. In *Ngn2*-derived neurons differentiated in the presence of LIF, specific activation of *vGLUT2* expression was observed on mRNA level together with a slight activation of *Th* (Fig. S6). On protein level, cells stained positive for vGLUT1 and NR1 (Fig. S7A-F). As a weak induction of *Th* mRNA was detectable, we also analysed TH protein expression. Very rarely, cells weakly expressing TH protein could be detected (Fig. S7G-I). These data indicate that *Ngn2*-induced differentiation preferably results in the appearance of glutamatergic neurons. However, the formation of other neuronal subtypes like dopaminergic neurons in the presence of LIF or of a mixed, artificial phenotype cannot be completely excluded.

### Functional Features of *Ngn2*-induced Neurons

**Figure 4:**
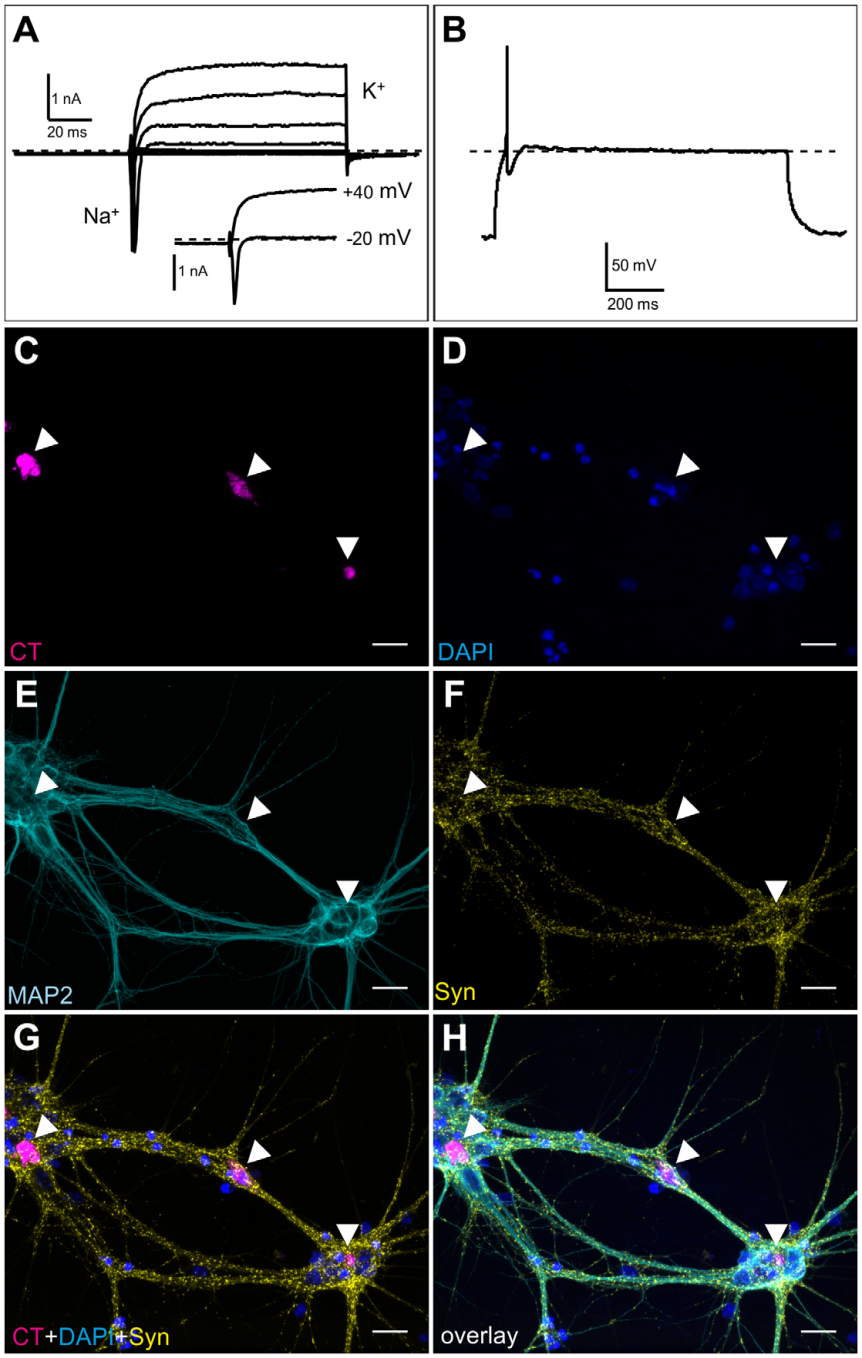
Functionality of *Ngn2*-derived neurons. (A, B) Whole-cell patch clamp recordings of neurons after *Ngn2*-induced neuronal differentiation 10 dpr in *Ngn2*-inducible cell line. Expression of *Ngn2* was induced by delivery of Cre recombinase as transducible protein. (A) Voltage-clamp recording upon application of depolarizing pulses ranging from −80 mV to +40 mV from a holding potential of −70 mV. Transient Na^+^ inward current (also: inset at −20 mV) were followed by sustained K+ outward current (inset: at +40 mV). (B) Current-clamp recording upon injection of a depolarizing pulse elicits a fast and high-amplitude action potential. (C-H) Contacts of *Ngn2*-induced neurons with primary hippocampal neurons at day 12 of co-culture and in total 20dpr. (C) CellTracker™ Green CMFDA (CT) labeled *Ngn2*-mESC derived neurons (magenta). (D) Nuclei visualized by DAPI staining (blue). (E) MAP2 staining (cyan). (F) Synapsin-1 staining (yellow). (G) Overlay of CT, DAPI, and synapsin-1 staining. (H) Overlay of C, D, E, F. Arrowheads mark the position of CT labeled *Ngn2-*mESC derived neurons. Scale bars: 20 µm.

To test if *Ngn2*-derived neurons show functional features of terminally differentiated neurons whole-cell patch-clamp recordings were performed. In the voltage-clamp mode voltage-gated currents typical for terminally differentiated neurons could be observed. Depolarizing pulses from a holding potential of −70 mV elicited fast-activating transient inward currents typical for voltage-activated Na^+^-currents (Na_v_ channels) followed by a delayed outward current indicative of voltage-activated potassium currents (K_v_ channels) (Fig. 4A). In the current-clamp mode injection of depolarizing current elicited an action potential with a duration of 5 ms and an amplitude of 105 mV (Fig. 4B).

Next, we wanted to investigate whether *Ngn2*-induced neurons can form contacts with primary neurons in co-culture. In co-culture with primary mouse hippocampal neurons, the E14-CreP2Angn2 cells expressed the presynaptic marker synapsin-1 and formed morphologically tight contacts with the hippocampal neurons (Fig. 4C-H).

### *Ngn2*-induced Neuronal Differentiation in Chemically Defined Media

Hitherto, our findings strongly suggest that *Ngn2* is sufficient to induce a neuronal differentiation process even under pluripotency promoting signals mediated by the combination of serum and LIF. To further strengthen this hypothesis, differentiation of E14-CreP2Angn2 cell line was performed in 2i medium. This chemically defined stem cell medium contains LIF, knock-out serum replacement and two small-molecule inhibitors that are able to maintain pluripotency and self-renewal of ESCs by the inhibition of defined signaling pathways [28]. Seven days post 4OHT-mediated induction of *Ngn2* expression, few Tuj1 positive neurons could be detected in 4OHT treated cultures. Mock treated cultures did not contain any Tuj1 positive cells (Fig. S8A, S8B, S8E, S8F). This indicates that *Ngn2* induces neuronal differentiation also in 2i medium conditions albeit at lower frequency compared to LIF and serum containing culture conditions (Fig. S8C, S8D). Thus, one can conclude that the induction of a neuronal fate by *Ngn2* is influenced by media conditions but does not depend on them. *Ngn2*-induced neuronal formation did not require any additional signals nor was it prevented by contradictory cues. This highlights the strength of the signal mediated by *Ngn2* and suggest that this might be beneficial in conventional differentiation protocols. To test this idea, E14-CreP2Angn2 cells were cultivated after *Ngn2* induction in N2B27 medium which is used in many neuronal differentiation protocols. Immunofluorescence staining for Tuj1 5dpr revealed that in these culture conditions neuronal cells were more frequent compared to –LIF conditions. Furthermore, already 5dpr, neurons cultured in N2B27 showed mature neuronal morphology and stained positive for Tau and Synapsin (Fig. S9). This indicates that *Ngn2*-induced differentiation is accelerated by the presence of a neuronal differentiation medium.

## Discussion

Exact cell fate determination is of crucial importance for correct embryogenesis. Numerous studies indicate that certain genes are essential for this process. However, it is still not clear if such genes can determine cell fate on their own, or they require a specific environment or auxiliary factors.

### *Ngn2* Induces Formation of Mature Neurons

In this study, we demonstrate that the bHLH transcription factor *Ngn2* is sufficient to induce efficient and rapid formation of mature neurons from pluripotent stem cells. These results indicate that certain genes have the potential to determine the cell fate choice of totally uncommitted stem cells outside the normal process of development. Several earlier studies report that ectopic expression of certain neuronal genes like *Ngn1*, *Ngn2*, or *Ascl1* can induce the formation of neural cells from different cell types [29], [30], [7], [4]. In these studies, ectopic expression of the fate-inducing genes was accompanied by changes of culture conditions like e.g. the addition of media components known to support the formation and survival of neuronal cells. As especially changes in medium compositions frequently have a considerable but often immeasurable influence on cellular processes it is difficult to evaluate the potential of single genes to determine cell fate decisions. Our findings prove that the cell fate defining potential of *Ngn2* is independent of additional external or internal signals.

The process of differentiation induced by *Ngn2* was confirmed by morphology, induction of early and late neural marker genes, expression of neuron-specific proteins, and electrophysiological activity unique to neurons. Furthermore, *Ngn2*-derived neurons formed contacts with hippocampal neurons in co-culture signifying their ability to interconnect with physiological neuronal networks. Although more in-depth analyses would be useful to fully determine the subtype as well as the status of functionality of the *Ngn2*-derived neurons, our data prove that *Ngn2* induces the formation of mature and at least partially functional neurons.

### *Ngn2*- induced Differentiation Shows Features of *in vivo* Neurogenesis

The gene expression pattern observed after *Ngn2* transfection revealed particularly interesting information regarding the mechanism of *Ngn2*-induced differentiation.

First, the upregulation of *Dcx* and *NeuN* indicates the progression of the differentiation process until the mature neuron stage. *Dcx* is a microtubule-associated protein that is transiently expressed in early postmitotic migrating neurons [21], [20]. Like *NeuN,* it is a marker for postmitotic neurons. NeuN is a nuclear protein that can be detected in terminally differentiated neurons throughout the nervous system [22].

Another important observation is the upregulation of *Math3* and *Olig2* during the differentiation process. *Math3* is expressed in the developing nervous system [15] and it has been shown that *in vivo Ngn2* and *Math3* are expressed in a temporal sequence with *Ngn2* expression preceding that of *Math3* [31]. Possibly, *Ngn2*-mediated differentiation *in vitro* follows at least partially its physiological genetic cascade. It has also been demonstrated that *in vivo* Math3 augments *Ngn2* activity [32]. Thus, one can speculate that in our *in vitro* experiment *Ngn2* does not induce unspecific neuronal differentiation, but activates defined differentiation pathways of *in vivo* neurogenesis with directed activation of its interaction partners.

This hypothesis is also supported by the observed upregulation of *Olig2* because *Ngn2* and *Olig2* have combinatorial roles in the generation of motor neurons [33], [34]. Interestingly, *Olig2,* together with the related *Olig1*, regulates expression of *Ngn2* in motor neurons [35] *in vivo*. This raises the question how and why *Olig2* expression was induced by ectopic *Ngn2* expression in our *in vitro* experiments.

Similarly, an interesting observation was the upregulation of *Sox1* and *Pax6*. *Sox1* is known to be the earliest marker of neural precursors and its expression precedes that of *Ngn2 in vivo* [14]. *Pax6* was shown to directly regulate *Ngn2* [36], [37]. In our study, both *Pax6* and *Sox1* were upregulated during *Ngn2*-induced differentiation processes, namely *Sox1* in transient transfection assays and *Pax6* after induction of *Ngn2* in a transgenic cell line.

It is surprising that three upstream genes, *Sox1, Pax6,* and *Olig2,* are activated in a differentiation process based on an inducing factor which, during physiological differentiation, is located genetically downstream in the cascade.

One hypothesis explaining this phenomenon would be that differentiating cells recapitulate the sequence of steps documented for neurogenesis *in vivo*, including stages that precede activation of the here employed inducing factor *Ngn2*. This idea is supported by a similar observation reported in another study describing cell conversion of medakafish spermatogonia into various somatic cell types by ectopic expression of lineage-specific TFs [38]. In that study, the activation of genes that *in vivo* are located upstream of the inducing factors was also detected for three different processes of cell fate conversion. Likewise, TF-induced differentiation of medakafish ESCs into melanocytes included the activation of upstream marker genes [39]. Thus, the here reported upregulation of *Sox1, Pax6,* and *Olig2* during *Ngn2*-mediated differentiation could be a conserved part of the *in vitro* differentiation process.

Alternatively, *Ngn2*-induced differentiation resulted in the formation of mature *Pax6* and *Olig2* positive neurons which is indicated by the increased expression of these genes in later stages of differentiation (Fig. 3A). This idea is in line with previous findings demonstrating that *Pax6* enhances the differentiation of neuroepithelial cells into radial glial cells and neurons [40]. *Olig2* has also been shown to be expressed in the adult brain albeit only in a small subpopulation of progenitor cells [41]. This is contradictory to the assumption that *Ngn2* expression led to the formation of mature neurons. However, it cannot be excluded that *Olig2* has an unknown function in postmitotic neurons that has still to be determined. Furthermore, *in vitro* generated neurons exist in an artificial environment that might induce phenotypical features – like an unusual gene expression - that are not displayed by neurons arising during physiological development.

Nevertheless, the gene expression pattern observed during *Ngn2*-mediated differentiation showed some similarities to processes of *in vivo* neurogenesis that are marked by *Ngn2* expression. These similarities included the activation of interaction partners and potential upstream regulators of *Ngn2*. Furthermore, the preference towards the formation of a glutamatergic phenotype is also reminiscent of *in vivo* corticogenesis where *Ngn1* and *Ngn2* specify glutamatergic cortical neurons [42]. The findings presented here indicate that *Ngn2* alone can activate parts of a genetic cascade in uncommitted stem cells. Further experiments will be required to test this idea and to analyse the underlying mechanisms. Nevertheless, these observations provide a new insight in the role of key developmental transcription factors in genetic networks.

### *Ngn2* Breaks Intra- and Extracellular Pluripotency Signals

Another important finding of this study is that *Ngn2* is able to induce and promote differentiation under conditions normally enhancing pluripotency. Cells were grown in medium with serum, which is normally omitted during in vitro neuronal differentiation protocols [43], [44] and in the presence of LIF. LIF was initially considered as a strong factor for the maintenance of pluripotency [45], [46] and has been shown to have an inhibitory effect on neural determination of stem cells [47]. Other studies, however, report that LIF enhances the differentiation of ESCs into neural progenitor cells albeit mainly as a permissive factor [43]. Thus, the role of LIF during the formation of neural cell types from ESCs is not definitely clear and probably depends on other environmental factors. In our study, addition of LIF was combined with conditions normally used to enhance the proliferation of undifferentiated stem cells. Our results prove that even under these conditions *Ngn2* expression leads to the loss of pluripotency markers like Nanog suggesting that the network maintaining pluripotency can be overcome by single defined signals. This idea is also strengthen by the fact that *Ngn2*-induced neuronal differentiation did also occur in chemically defined 2i medium. Thus, although these pluripotency promoting conditions affected the efficiency of the *Ngn2*-induced differentiation process, they were not sufficient to prevent it. This proves that the signal mediated by *Ngn2* does not depend on extracellular cues.

Interestingly, our data indicate that not all cells that express ectopic *Ngn2* differentiate into neuronal cells. Gene and protein expression analyses suggest that these non-responsive cells are *Nanog* and *Oct4* positive and –in the presence of LIF – form stem-cell like colonies. Thus, *Ngn2*-expressing cells either undergo a neuronal differentiation process or retain features of pluripotent stem cells. One possible explanation for this varying responsiveness to *Ngn2* could be the heterogeneity of mESC cultures. Indeed, it has been shown that mESCs oscillate between various states that are more or less prone to various differentiation cues [26], [48].These oscillations could influence the ability of mESCs to undergo a differentiation process upon *Ngn2* expression.

Altogether, the here demonstrated potential of *Ngn2* to define cell fate decisions *in vitro* is in line with the dominant role of *Ngn2* during neurogenesis *in vivo*. During mouse embryogenesis, *Ngn2* expression is detected widely in the developing CNS and PNS [49] and *Ngn2* knockout mice exhibit severe neural defects and die shortly after birth [31]. Interestingly, it has been shown that the *Ngn2*-related *Ngn1* can specify a neural fate in a non-physiological context in zebrafish embryos [50]. Although it cannot be excluded that in that study the effects of *Ngn1* were influenced by unknown factors of the *in vivo* environment one can assume that *Neurogenins* have the ability to define a neuronal identity in non-neuronal cells. This hypothesis is confirmed by our data demonstrating that *Ngn2* can induce and promote a complete neuronal differentiation process.

### Conclusion

In summary, our data prove that ectopic expression of *Ngn2* is sufficient to induce the formation of mature neurons from stem cells. This is, to our knowledge, the first study reporting that a single TF determines the fate of totally uncommitted stem cells without the need for additional signals and independent of culture conditions.

TF-induced differentiation therefore constitutes a promising alternative or expansion to conventional differentiation protocols. *Ngn2*-mediated differentiation is fast and robust and furthermore differs from standard differentiation protocols in the fact that both induction of differentiation and lineage commitment depend on a single factor and are totally independent of external signals. Therefore, this system is less susceptible to variability compared to approaches depending on several parameters which each can have unexpected effects. Thus, single gene mediated differentiation lends itself to a model for differentiation studies that require a very robust and reproducible differentiation process. Alternatively, single gene mediated differentiation can be combined with medium-based strategies to improve rapidness, efficiency, and the levels of control over the direction of differentiation.

In addition, our findings are a proof of concept for the feasibility of single gene mediated differentiation. We suggest that this approach can be extended to generate other cell types if the appropriate TFs are identified. Indeed, we were recently able to use this approach for the generation of myoblasts by ectopic expression of *MyoD1* [51]. Importantly, this process could be combined with the here presented *Ngn2*-induced differentiation allowing the formation of neurons and myoblasts in parallel. Thus, the concept of single gene mediated differentiation enables the simultaneous generation of unrelated cell types as mixed cultures.

Moreover, the here presented system provides a valuable tool for studies of neural development, the loss of pluripotency, and effects of key developmental genes on cell fate decisions.

## Supporting Information

Figure S1 **Induction of neuronal differentiation by transient transfection with** ***Ngn2*** **in the absence of LIF.** (A-D) 5dpt, *Ngn2*-transfected cells display neuronal morphology and express neuronal marker proteins like Tuj1 (A,B) and Map2ab (C,D). (B,D) Overlays of immunofluorescence staining and Hoechst staining. Scale bars: 20 µm. (E-J) Loss of Nanog expression (arrowheads) 3dpt in *Ngn2*-transfected (E,G,I), but not in mock-transfected cells (F,H,J). Transfected cells are visualized by expression of cotransfected GFP (E,F). Scale bars: 20 µm. (K) Gene expression pattern of untreated (ut), *Ngn2*-transfected (d5+, d7+), and mock-transfected (d5-, d7-) mESCs 5 and 7dpt. b: Brain cDNA. Dashed lines indicate grouping of different parts from the same gel. A representative result from three independent experiments is shown. (L) Tuj1 positive cells in *Ngn2*-transfected and mock-transfected cells 5 and 7dpt. Absolute numbers are shown as non-differentiating cells continue proliferating. Therefore, the relative number would not really reflect the increase of neurons upon *Ngn2* compared to mock transfection. Columns show mean +/−SD of three independent experiments.
(TIF)

Figure S2 **Expression of ectopic** ***Ngn2*** **specifically in developing neurons differentiated in the absence (A-C) or the presence (D-E) of LIF.** Immunofluorescence staining for myc-tagged Ngn2 (A, D) and Tuj1 (B, E) in *Ngn2*-transfected mESCs 5dpt Overlays (C, F) reveal that neurons express ectopic *Ngn2*. Scale bars represent 20 µm.
(TIF)

Figure S3 **STAT3 immunofluorescence staining proving active LIF signaling.** (A) Colony of non-transfected mESCs treated for 24 hours with conditioned medium from *Ngn2*-transfected cells. (B) STAT3 staining. (C) Nuclei visualized by Hoechst staining. (D) Overlay of B and C showing nuclear localization of STAT3. Scale bars: 20 µm.
(TIF)

Figure S4 **Loss of Nanog protein expression in E14-CreP2Angn2 cells upon induction of** ***Ngn2*** **expression by 4OHT treatment.** 4OHT (D-F) and mock treated cells (A-C) 3dpr in the presence of LIF. 4OHT (J-L) and mock treated cells (G-I) 3dpr in the absence of LIF. Scale bars: 20 µm.
(TIF)

Figure S5 **Neuronal differentiation in E14-CreP2Angn2 cells.** (A) Efficiency of neuron formation 7 days post recombination in the presence (16.7%) and absence of LIF (41.8%). Columns show mean +/−SD of three independent experiments. (B-D) ESC-like colonies (arrow) remaining in *Ngn2*-expressing cultures in the presence of LIF. Immunofluorescence of E14-CreP2Angn2 cells 7dpr for Nanog (B) and Tuj1 (C). (D) Overlay showing Nanog+/Tuj1- cells. Scale bars: 50 µm. (E) Expression of non-ectodermal differentiation and of pluripotency markers in 4OHT (d7+) treated and mock treated (d7-) E14-CreP2Angn2 cells 7dpr differentiated in the presence or in the absence of LIF. A representative result from three independent experiments is shown. (ut) untreated. (+) positive control.
(TIF)

Figure S6 **Neuronal marker expression of E14-CreP2Angn2 derived neurons differentiated in the presence of LIF.** (ut) untreated, (d7+) 4OHT treated, (d7-) mock treated, (b) Brain cDNA. Dashed lines indicate grouping of different parts from the same gel. A representative result from three independent experiments is shown.
(TIF)

Figure S7 **Neuronal differentiation of E14-CreP2Angn2 cell line in the presence of LIF.** Expression of vGLUT1 (A-C) and NR1 (D-F) indicating the formation of glutamatergic neurons. (G-I) Very rarely, cells positive for TH (arrows) could be detected. Scale bars: 20 µm (A-C), 10 µm (D-F), 50 µm (G-I).
(TIF)

Figure S8 **Neuronal differentiation of E14-CreP2Angn2 cells in chemically defined 2i medium.** Overlays of Tuj1 and Hoechst staining 7dpr. (A, B) Induction of *Ngn2* results in neuron formation in 2i medium (A, arrowheads) with no neurons detectable in mock-treated cells (B). (C, D) *Ngn2*-induced neuron formation is more efficient in LIF and serum containing medium. (E, F) Close-up of representative Tuj1 positive neuronal cells detected 7 days post recombination in 2i medium condition. Scale bars: 100 µm (A-D), 20 µm (E, F).
(TIF)

Figure S9 **Enhanced neuronal differentiation of E14-CreP2Angn2 cells in neuronal differentiation medium N2B27.** (A, B) Wide field scans of Tuj1 staining of cells differentiated in LIF-free ESC medium (A) or N2B27 (B) 5dpr. Images were taken with exposure time and gain settings. Neurons formed under N2B27 culture conditions are more frequent and show a more mature phenotype. Scale bars: 200 µm. (C, D) Close-ups of neurons differentiated in N2B27 medium 5dpr. Cells show morphology of mature neurons and stain positive for Tau and Synapsin. Scale bars: 20 µm.
(TIF)
