# Supplementary figures and images for: Ectopic Expression of Neurogenin 2 Alone is Sufficient to Induce Differentiation of Embryonic Stem Cells into Mature Neurons
Source: PLoS One. 2012 Jun 13;7(6):e38651. doi: 10.1371/journal.pone.0038651 (PMC3374837; doi:10.1371/journal.pone.0038651)

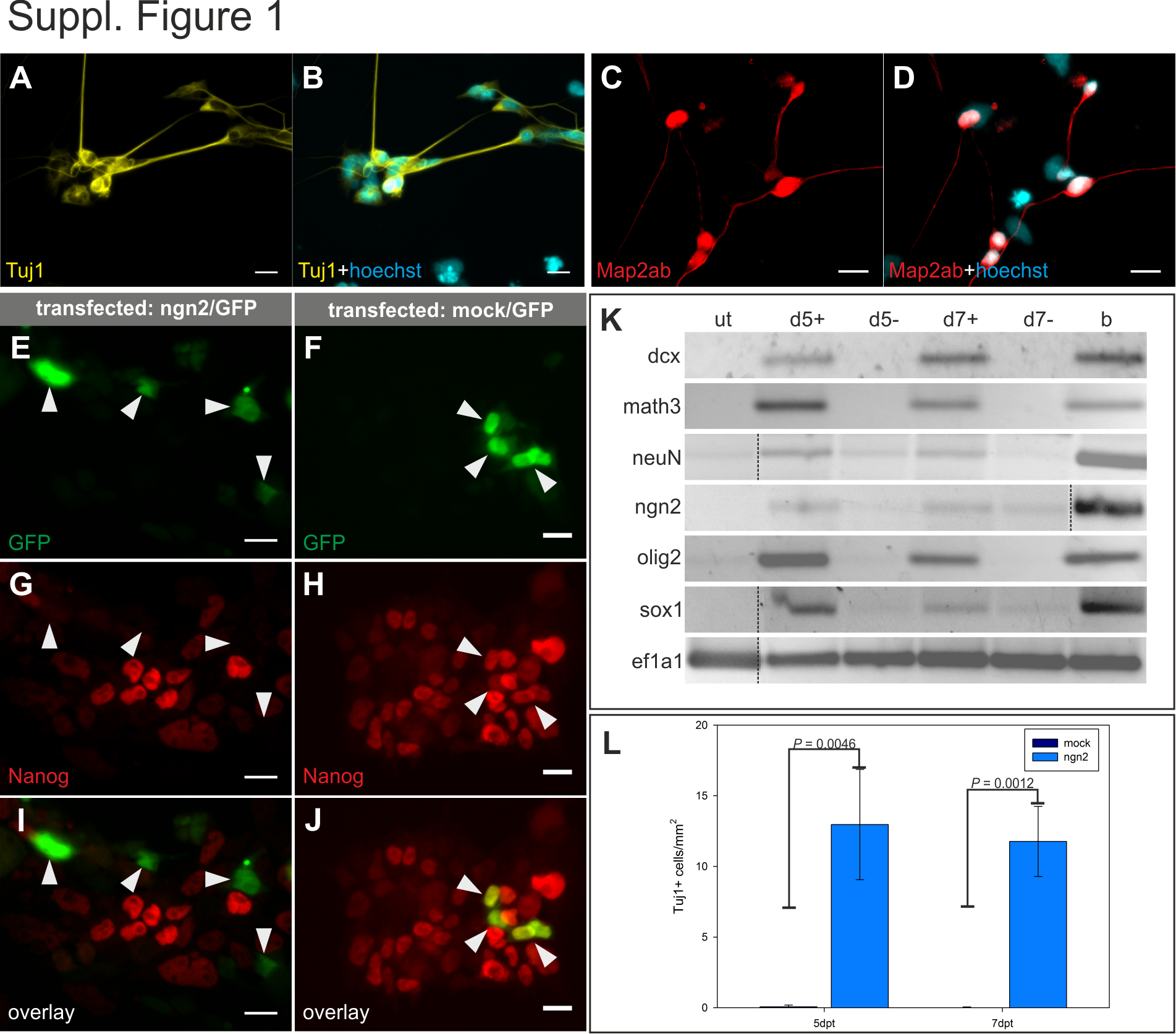

Supplement: Figure S1 — Induction of neuronal differentiation by transient transfection with Ngn2 in the absence of LIF. (A-D) 5dpt, Ngn2-transfected cells display neuronal morphology and express neuronal marker proteins like Tuj1 (A,B) and Map2ab (C,D). (B,D) Overlays of immunofluorescence staining and Hoechst staining. Scale bars: 20 µm. (E-J) Loss of Nanog expression (arrowheads) 3dpt in Ngn2-transfected (E,G,I), but not in mock-transfected cells (F,H,J). Transfected cells are visualized by expression of cotransfected GFP (E,F). Scale bars: 20 µm. (K) Gene expression pattern of untreated (ut), Ngn2-transfected (d5+, d7+), and mock-transfected (d5-, d7-) mESCs 5 and 7dpt. b: Brain cDNA. Dashed lines indicate grouping of different parts from the same gel. A representative result from three independent experiments is shown. (L) Tuj1 positive cells in Ngn2-transfected and mock-transfected cells 5 and 7dpt. Absolute numbers are shown as non-differentiating cells continue proliferating. Therefore, the relative number would not really reflect the increase of neurons upon Ngn2 compared to mock transfection. Columns show mean +/−SD of three independent experiments. (TIF) [file pone.0038651.s001.tif]

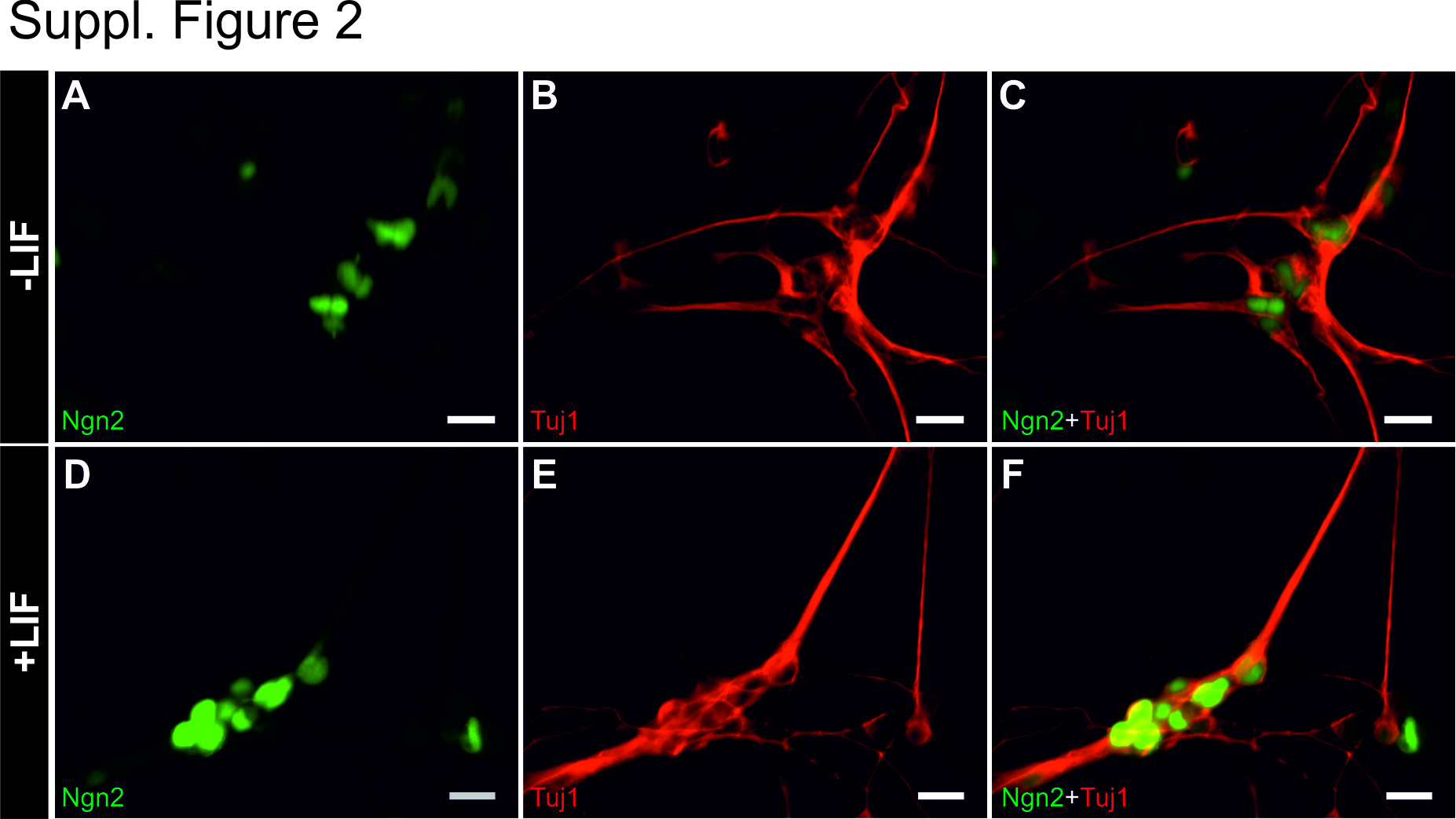

Supplement: Figure S2 — Expression of ectopic Ngn2 specifically in developing neurons differentiated in the absence (A-C) or the presence (D-E) of LIF. Immunofluorescence staining for myc-tagged Ngn2 (A, D) and Tuj1 (B, E) in Ngn2-transfected mESCs 5dpt Overlays (C, F) reveal that neurons express ectopic Ngn2. Scale bars represent 20 µm. (TIF) [file pone.0038651.s002.tif]

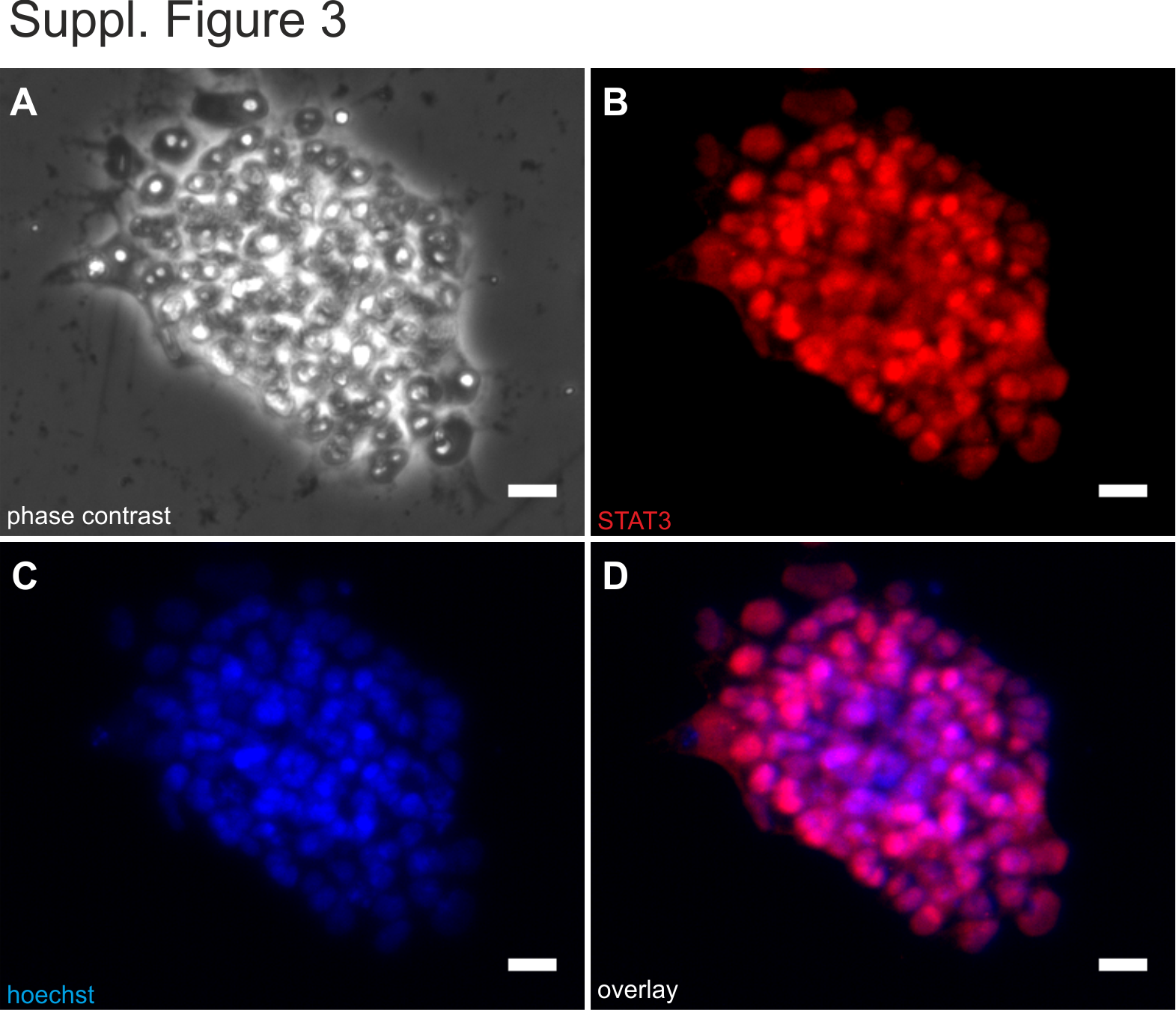

Supplement: Figure S3 — STAT3 immunofluorescence staining proving active LIF signaling. (A) Colony of non-transfected mESCs treated for 24 hours with conditioned medium from Ngn2-transfected cells. (B) STAT3 staining. (C) Nuclei visualized by Hoechst staining. (D) Overlay of B and C showing nuclear localization of STAT3. Scale bars: 20 µm. (TIF) [file pone.0038651.s003.tif]

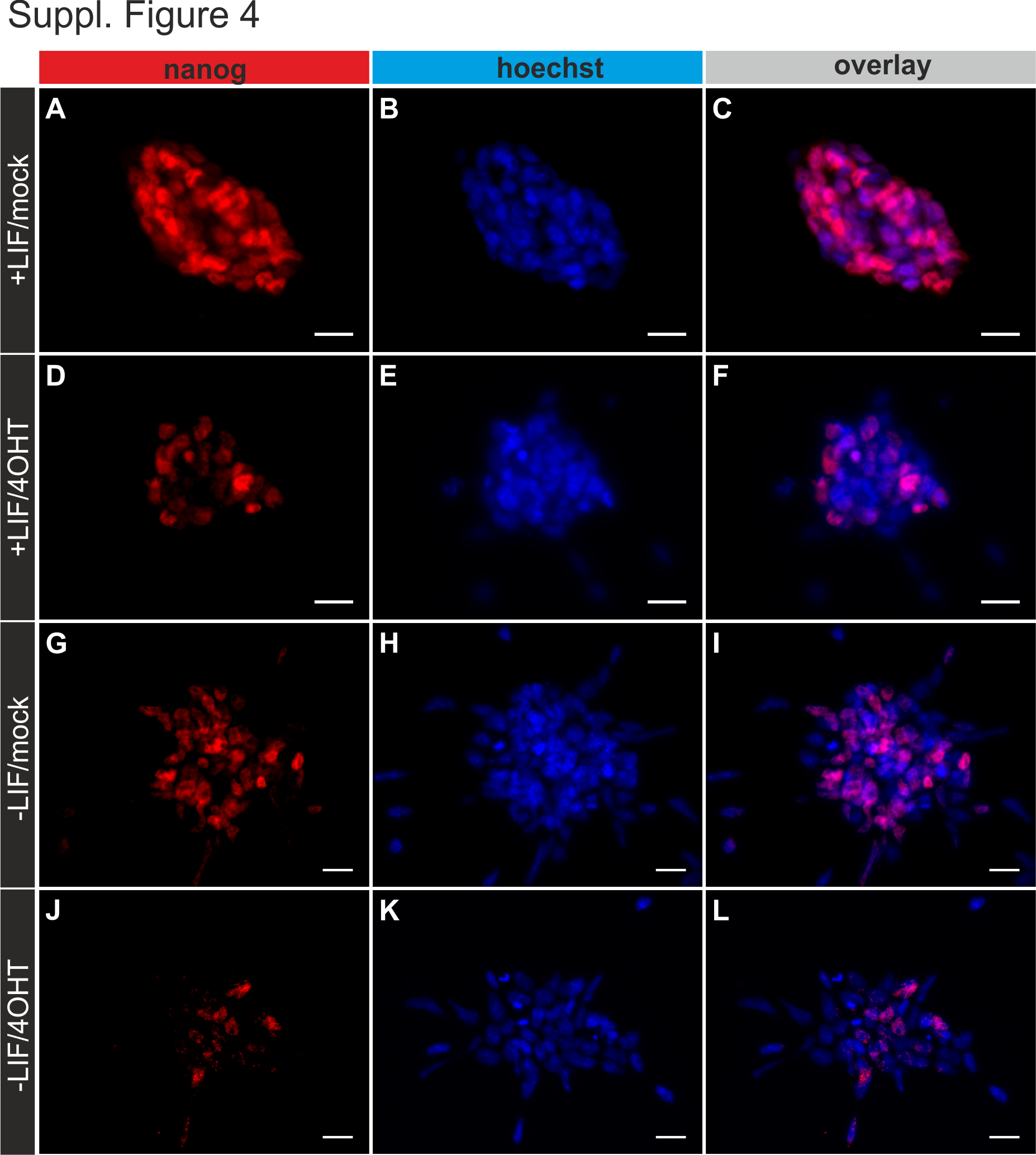

Supplement: Figure S4 — Loss of Nanog protein expression in E14-CreP2Angn2 cells upon induction of Ngn2 expression by 4OHT treatment. 4OHT (D-F) and mock treated cells (A-C) 3dpr in the presence of LIF. 4OHT (J-L) and mock treated cells (G-I) 3dpr in the absence of LIF. Scale bars: 20 µm. (TIF) [file pone.0038651.s004.tif]

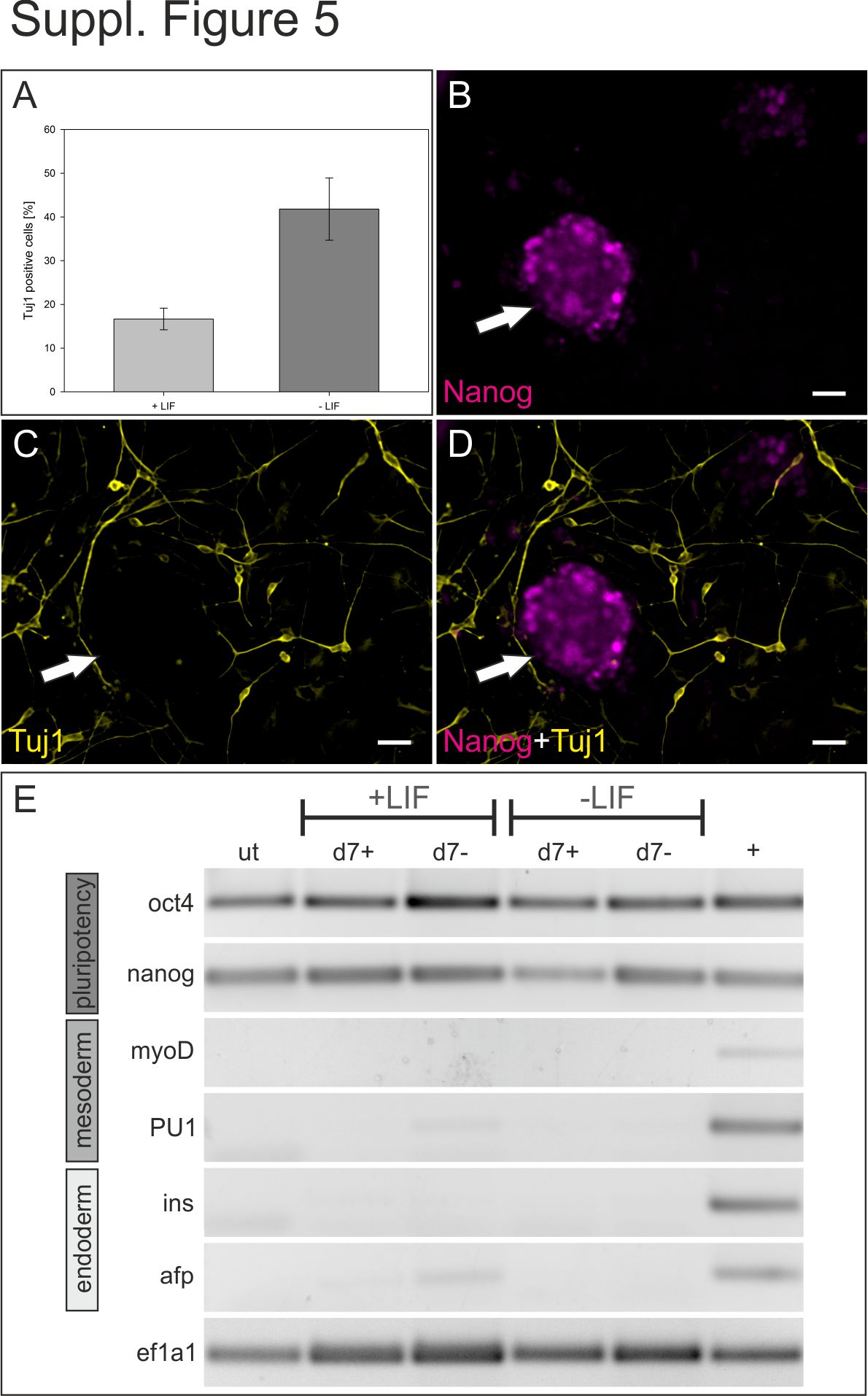

Supplement: Figure S5 — Neuronal differentiation in E14-CreP2Angn2 cells. (A) Efficiency of neuron formation 7 days post recombination in the presence (16.7%) and absence of LIF (41.8%). Columns show mean +/−SD of three independent experiments. (B-D) ESC-like colonies (arrow) remaining in Ngn2-expressing cultures in the presence of LIF. Immunofluorescence of E14-CreP2Angn2 cells 7dpr for Nanog (B) and Tuj1 (C). (D) Overlay showing Nanog+/Tuj1- cells. Scale bars: 50 µm. (E) Expression of non-ectodermal differentiation and of pluripotency markers in 4OHT (d7+) treated and mock treated (d7-) E14-CreP2Angn2 cells 7dpr differentiated in the presence or in the absence of LIF. A representative result from three independent experiments is shown. (ut) untreated. (+) positive control. (TIF) [file pone.0038651.s005.tif]

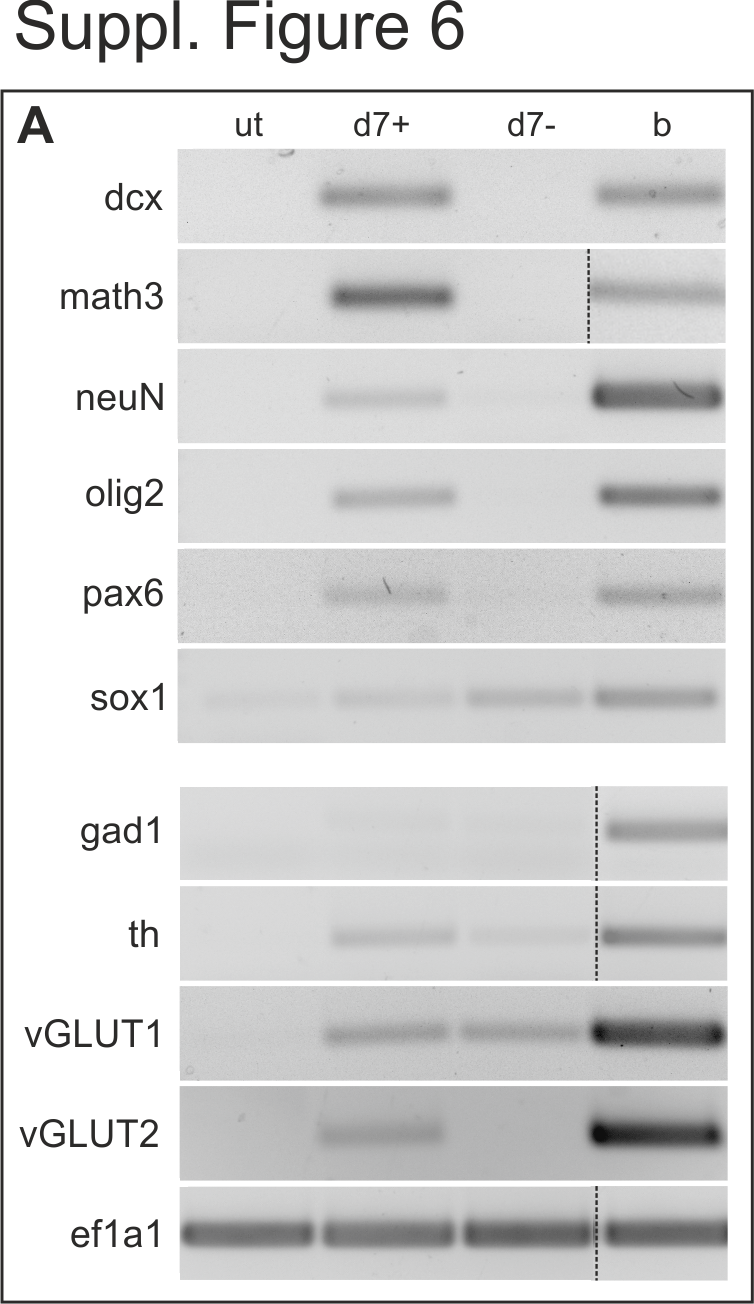

Supplement: Figure S6 — Neuronal marker expression of E14-CreP2Angn2 derived neurons differentiated in the presence of LIF. (ut) untreated, (d7+) 4OHT treated, (d7-) mock treated, (b) Brain cDNA. Dashed lines indicate grouping of different parts from the same gel. A representative result from three independent experiments is shown. (TIF) [file pone.0038651.s006.tif]

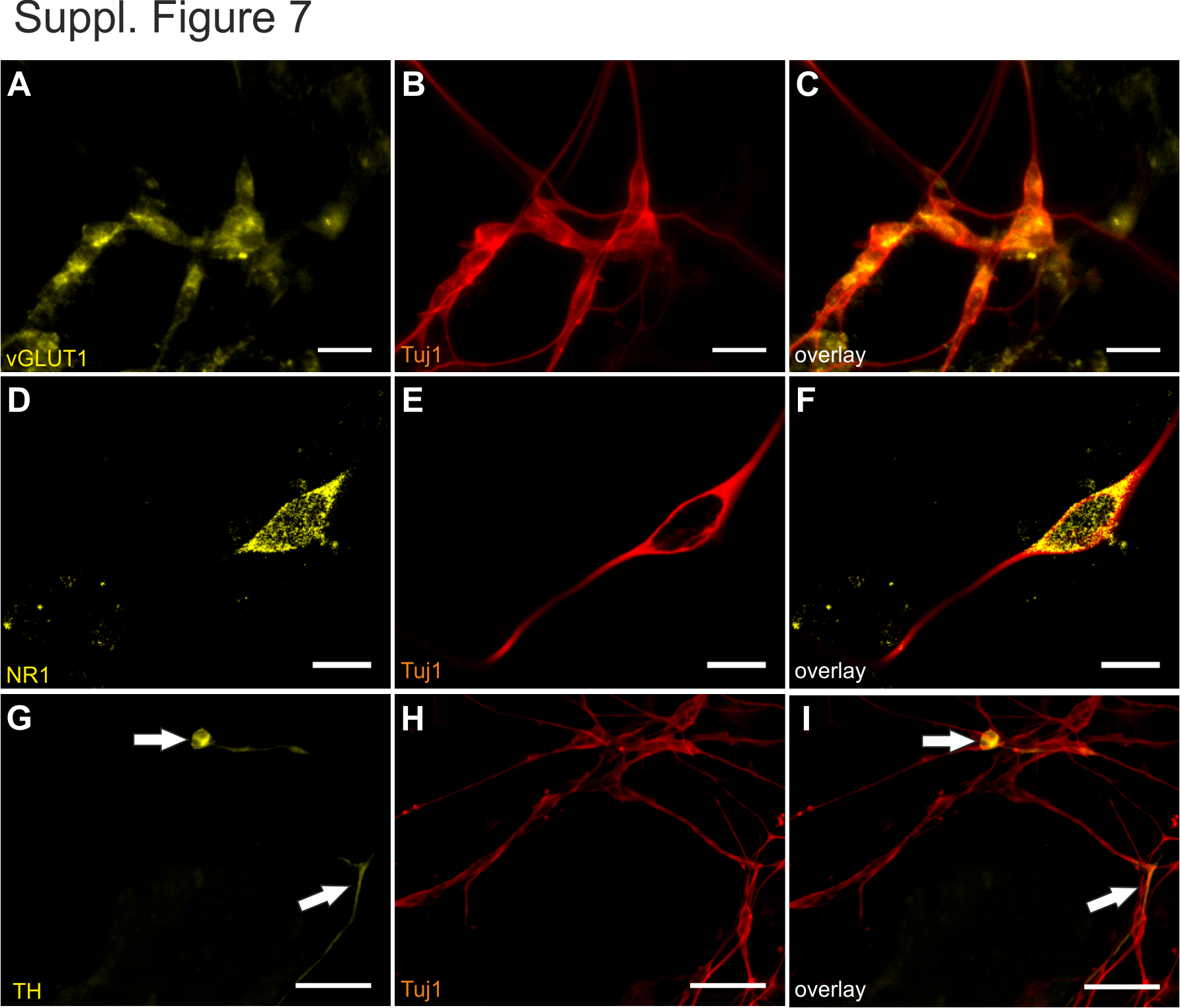

Supplement: Figure S7 — Neuronal differentiation of E14-CreP2Angn2 cell line in the presence of LIF. Expression of vGLUT1 (A-C) and NR1 (D-F) indicating the formation of glutamatergic neurons. (G-I) Very rarely, cells positive for TH (arrows) could be detected. Scale bars: 20 µm (A-C), 10 µm (D-F), 50 µm (G-I). (TIF) [file pone.0038651.s007.tif]

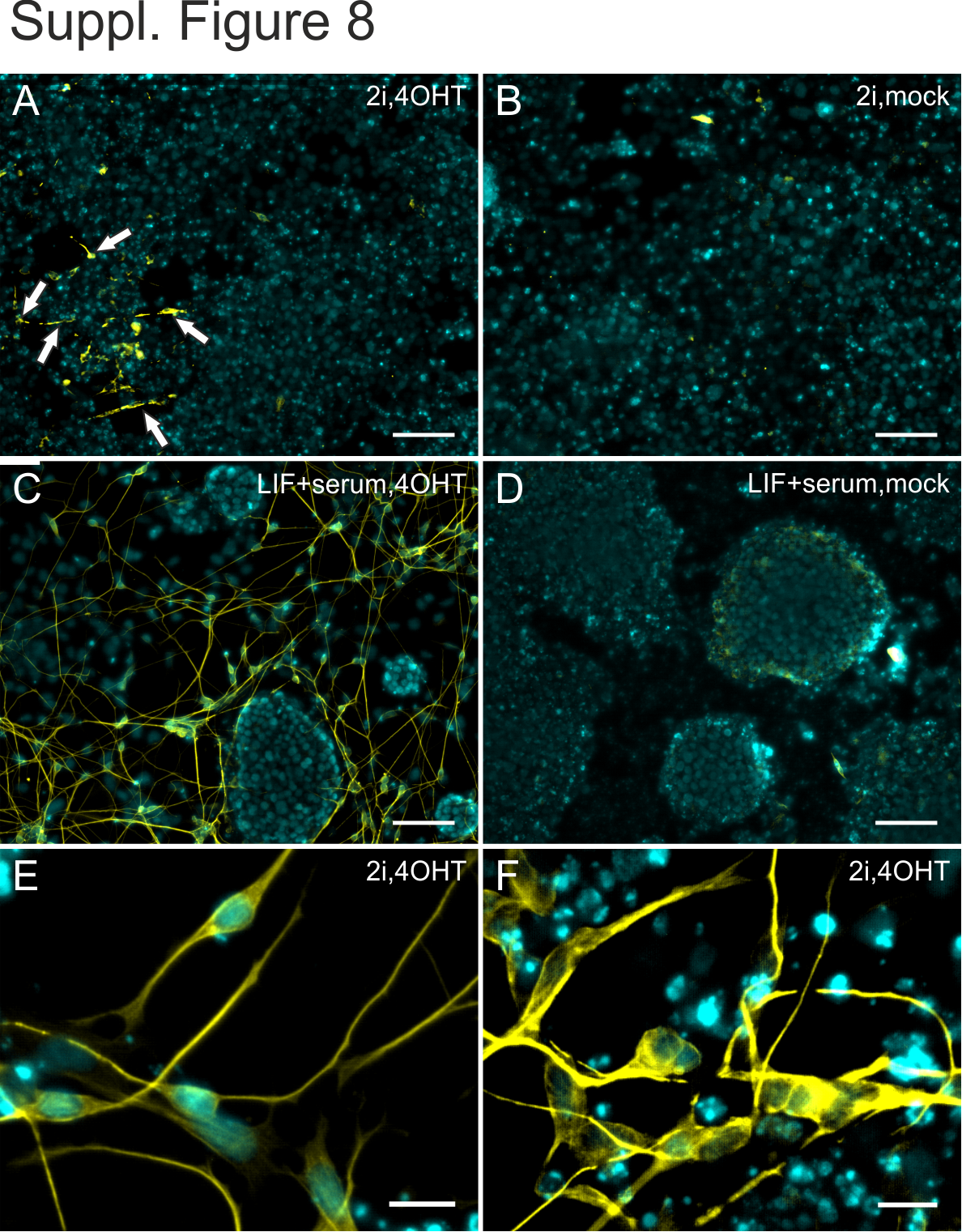

Supplement: Figure S8 — Neuronal differentiation of E14-CreP2Angn2 cells in chemically defined 2i medium. Overlays of Tuj1 and Hoechst staining 7dpr. (A, B) Induction of Ngn2 results in neuron formation in 2i medium (A, arrowheads) with no neurons detectable in mock-treated cells (B). (C, D) Ngn2-induced neuron formation is more efficient in LIF and serum containing medium. (E, F) Close-up of representative Tuj1 positive neuronal cells detected 7 days post recombination in 2i medium condition. Scale bars: 100 µm (A-D), 20 µm (E, F). (TIF) [file pone.0038651.s008.tif]

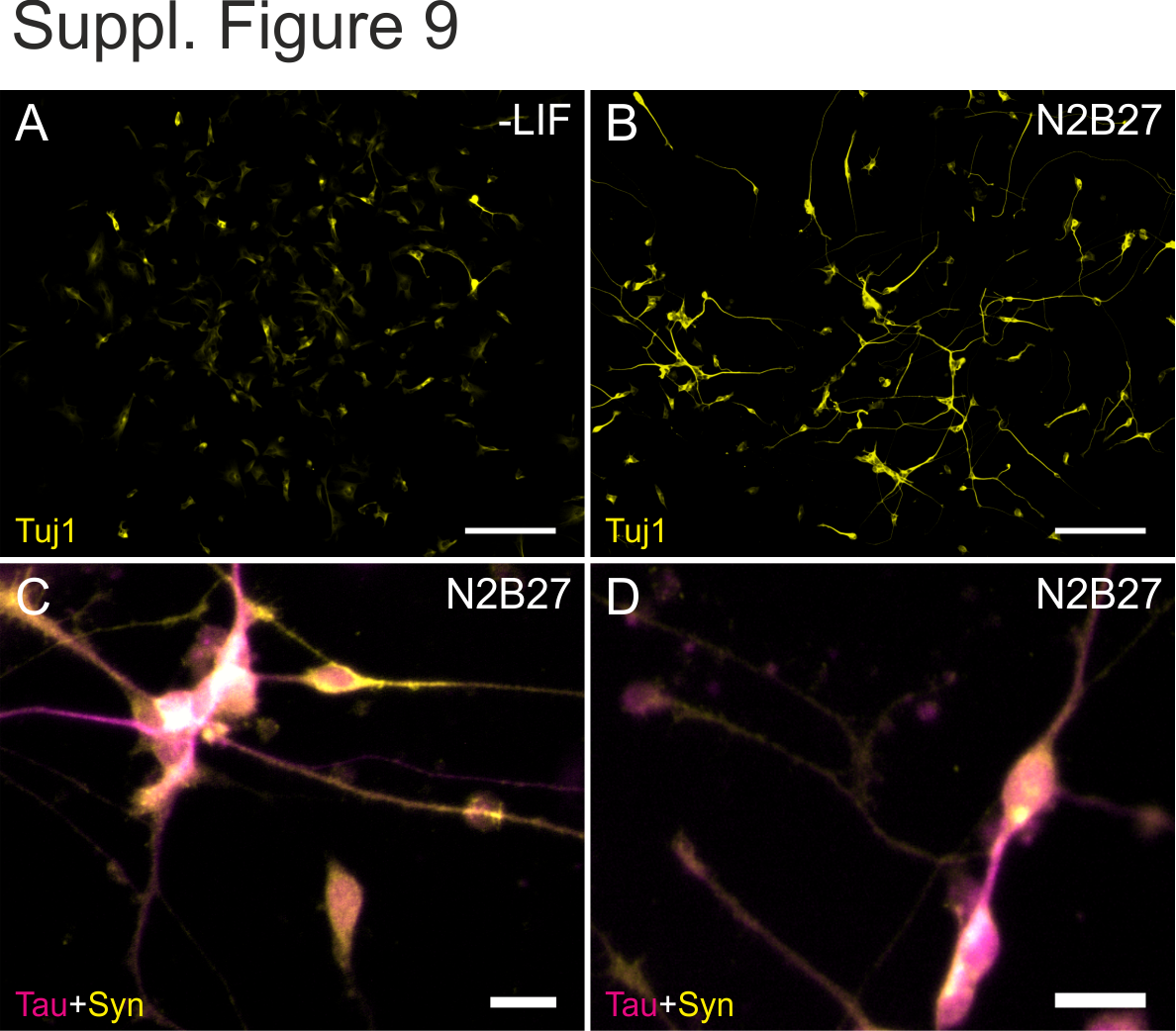

Supplement: Figure S9 — Enhanced neuronal differentiation of E14-CreP2Angn2 cells in neuronal differentiation medium N2B27. (A, B) Wide field scans of Tuj1 staining of cells differentiated in LIF-free ESC medium (A) or N2B27 (B) 5dpr. Images were taken with exposure time and gain settings. Neurons formed under N2B27 culture conditions are more frequent and show a more mature phenotype. Scale bars: 200 µm. (C, D) Close-ups of neurons differentiated in N2B27 medium 5dpr. Cells show morphology of mature neurons and stain positive for Tau and Synapsin. Scale bars: 20 µm. (TIF) [file pone.0038651.s009.tif]
